# Repeatability of Selected Parameters Related to Stallion Sperm Quality and Cryotolerance

**DOI:** 10.3390/ani15192805

**Published:** 2025-09-26

**Authors:** Raffaele Boni, Raffaella Ruggiero, Felisia De Luca, Maria Lucia Serritella, Tommaso Di Palma, Stefano Cecchini Gualandi

**Affiliations:** Department of Basic and Applied Sciences (DiSBA), University of Basilicata, Via dell’Ateneo Lucano, 10, 85100 Potenza, Italyfelisia.deluca@studenti.unibas.it (F.D.L.); stefano.cecchini@unibas.it (S.C.G.)

**Keywords:** equine, freezability, sperm kinetics, mitochondrial membrane potential, lipid peroxidation, ROS, nitric oxide, stallion age

## Abstract

This study evaluated the repeatability of selected sperm parameters in Salernitano stallions housed on the same farm. Semen was collected weekly for four weeks, and sperm kinetics, mitochondrial activity, and oxidative/nitrosative status were assessed before and after freezing the sperm with HF-20 and INRA Freeze. Pre-freezing, significant individual variability was observed, with low repeatability for semen volume (*r* = 0.32), total motility (*r* = 0.38), curvilinear velocity (*r* = 0.32), and lipoperoxidation (*r* = 0.36). Post-thaw, sperm frozen with INRA Freeze showed significant inter-stallion differences and low-to-moderate repeatability across kinematic parameters, mitochondrial membrane potential, nitric oxide, and lipoperoxidation, whereas those frozen with HF-20 showed repeatability only for progressive motility and intracellular H_2_O_2_. An assessment of freezability revealed significant inter-stallion variability and low-to-moderate repeatability for most kinematic traits in sperm frozen with INRA Freeze. Age influenced specific parameters in both fresh and frozen–thawed semen. Kinematic traits were strongly intercorrelated and associated with mitochondrial activity, as well as with lipoperoxidation, the latter being significantly related to H_2_O_2_ and nitric oxide levels. Although the overall post-thaw differences between extenders were not statistically significant, INRA Freeze enabled clearer discrimination among stallions. The generally low-to-moderate repeatability observed in this study suggests that extender choice can influence cryopreservation outcomes, and supports the need for tailored protocols.

## Simple Summary

Numerous studies have shown individual differences in sperm sensitivity to cryopreservation in horses. However, it remains unclear whether these individual responses are consistent over time. To investigate this, semen was collected weekly from a group of Salernitano stallions bred and housed on the same farm. Samples were assessed before and after freezing for kinematic parameters, mitochondrial function, and oxidative/nitrosative stress markers using two freezing extenders: HF-20 and INRA Freeze. Significant differences among stallions were observed in semen volume, some sperm motility parameters, and lipid peroxidation levels before freezing. Post-thaw analysis also revealed individual variations in sperm kinetics, reactive oxygen and nitrogen species, and lipid peroxidation, with low-to-moderate repeatability, especially in sperm frozen with INRA Freeze. A comparison of pre- and post-thaw data further confirmed consistent individual differences in freezability, which were again more evident in sperm frozen with INRA Freeze. Although no statistically significant differences were found between the two freezing extenders overall, INRA Freeze allowed for better discrimination between stallions. Age influenced certain sperm parameters in semen, as well as in both fresh and frozen–thawed sperm. These results emphasize the importance of extender choice in the assessment of sperm freezability, and support the development of individualized cryopreservation strategies.

## 1. Introduction

Sperm cryopreservation in stallions is becoming an increasingly established technique; however, it continues to face significant inefficiencies, primarily due to variability among individuals in terms of their sperm’s resilience to cryogenic stress [1]. This resilience, known as cryotolerance or freezability, can be estimated by comparing the functional parameters of sperm before and after they have undergone the freezing process. Although many studies have classified stallions as either “good” or “poor” freezers [2,3,4], there is currently no universally accepted criterion for defining freezability. This lack of standardization limits its practical use in the selection of stallions for breeding and impedes research into the factors—beyond individual variation—that may influence cryotolerance. Ebel and colleagues [5] proposed a freezability index based on the reduction observed in sperm viability after thawing compared to pre-freezing levels as a potential criterion for distinguishing between good and poor freezers. Unlike other sperm parameters, including total motility, this index was able to differentiate stallions’ freezability between the breeding and non-breeding seasons. However, assessing sperm viability often requires specific markers and specialized equipment, which are not always available under field conditions. Therefore, extending the investigation to include additional sperm kinematic parameters would be advantageous, as sperm kinetics is typically strongly correlated with viability [6,7] and can be readily assessed on-site [8]. Accordingly, sperm kinematic parameters could serve as practical and accessible alternatives for evaluating sperm freezability.

To address the issue of poor freezability in some stallions, commercial companies have developed customized freezing extenders. The use of such extenders is most effective when poor freezability is a consistent trait in an individual, rather than a transient condition influenced by fluctuating or temporary environmental factors, as demonstrated by Janet et al. [9], who found seasonal variations to affect both sperm quality and freezability. An analysis of the repeatability of parameters related to semen quality could clarify whether individual variability in response to freezing is a condition that persists over time in the same subject, as well as clarifying the potential leeway afforded by the use of customized freezing extenders.

Repeatability is the proportion of the total variance in multiple measurements of a trait that is due to differences among individuals. It is a useful tool for quantifying the extent to which an individual’s performance or behavior remains consistent over time, and can set the upper limit to heritability [10]. Generally, repeatability can be classified as low, moderate, or high if the value is <0.4, is between 0.4 and 0.7, or is >0.7, respectively [11]. In Lusitano stallions, using an Animal Model for genetic analysis, the estimated heritability (h^2^) (and repeatability, *r*) of various semen variables was 0.27 (0.35) for gel-free volume, 0.02 (0.38) for sperm concentration, 0.24 (0.44) for sperm motility, 0.29 (0.39) for total number of spermatozoa, and 0.41 (0.41) for total number of motile spermatozoa per ejaculate [12]. In German Warmblood stallions, Gottschalk and colleagues [13] estimated that h^2^ = 0.28 for gel-free semen volume, 0.21 for sperm concentration, 0.14 for sperm motility, 0.14 for the total number of spermatozoa, and 0.13 for the total number of motile sperm. Later, in frozen–thawed stallion sperm, the same team of researchers [14] estimated that h^2^ = 0.45 for the DNA fragmentation index, 0.13 for progressive motility, and 0.11 for non-viable sperm counts. These results suggest that it is possible to improve semen quality through selection, and that some properties of the semen produced by a stallion tend to remain consistent throughout the animal’s lifetime.

This study aimed to evaluate the repeatability of selected sperm physiological parameters related to sperm kinetics, bioenergetics and oxidative/nitrosative stress markers across multiple semen samples collected from Salernitano stallions. These parameters were evaluated before and after freezing the sperm using two different extenders: a commercial extender with a patented, undisclosed composition, and another extender with a known formulation that was prepared in our laboratory [15]. In addition, by comparing each parameter before and after freezing, new freezability indices were developed and analyzed.

## 2. Materials and Methods

### 2.1. Materials

Unless otherwise indicated, all the materials used for this study were purchased from Merck Life Science (Milan, Italy) and were cell culture-tested.

### 2.2. Animal Collection and Breeding Care

From February to May 2025 (late winter to spring), four ejaculates were collected weekly from each of eleven stallions used for semen production. These animals were of the Salernitano breed, had ages ranging from 4 to 18 years old (11.2 ± 5.9 years), and were of proven fertility. All the stallions were clinically healthy and were fed a standard diet consisting of approximately 10 kg of mixed hay and 2–2.5 kg of basic concentrate (on a dry-matter basis: crude protein 13.5%, fat 4.0%, crude fiber 6.8%), without antioxidant supplementation, and had ad libitum access to water. They were housed in individual paddocks at the Regional Center of Equine Improvement (Centro Regionale di Incremento Ippico-S. Maria Capua Vetere, Caserta, Italy). This center is approved for equine semen collection by the Regional Government of Campania, Italy (authorization number: U 1500083 CE000642004), and operates under rigorous health and animal welfare protocols.

All the animal procedures conducted complied with the European Union guidelines (Directive 2010/63/EU and D. Lgs. 4/03/2014 n. 26) on the protection of animals used for scientific purposes, and focus was placed on minimizing the number of animals used and reducing any pain or stress.

### 2.3. Experimental Design

Semen was collected once weekly from each of the eleven stallions, with the collection proceeding in groups of two or three stallions at a time and being repeated up to the fourth sampling before moving on to a new group of stallions. During the study period, no additional semen collections were performed outside of the trial. The collected samples were grossly evaluated for volume, sperm concentration, and motility. Then, the semen was diluted 1:2 and subjected to a gradual decrease in temperature during its transport to the laboratory. There, the semen samples were analyzed for sperm concentration, motility, and selected physiological parameters for assessing sperm bioenergetics—mitochondrial membrane potential, MMP—and oxidative/nitrosative markers—hydrogen peroxide (H_2_O_2_) and nitric oxide (NO) contents and lipoperoxidation (LPO). Meanwhile, the semen was diluted using two different freezing extenders and subsequently frozen. After thawing, sperm motility and physiological parameters were re-evaluated using the same methods as used before freezing. Freezability was assessed by comparing the post-thaw values of kinematic and physiological parameters with their corresponding pre-freezing values. The repeatability of each measured variable was then estimated.

### 2.4. Sperm Collection, Dilution, and Shipping

Semen collection was performed following exposure to a teaser mare using a Missouri artificial vagina fitted with an in-line sterile gauze to filter out the gel fraction of the ejaculate. Before sampling, each stallion underwent at least three preliminary semen collections to empty the sperm epididymal reservoir. Each ejaculate gel-free volume was evaluated using a 20 mL syringe, and then diluted 1:2 (volume of semen to volume of extender) with INRA 96^®^ (DLM Meazza, Lodi, Italy) [16] that was heated to 37 °C before semen collection. The tubes containing diluted sperm were placed in a jar filled with one liter of water at 37 °C, which was positioned inside a polystyrene box together with frozen eutectic plates placed around the outside of the jar. This setup allowed the sample temperature to progressively decrease to room temperature (RT = 20 °C) within the shipping period (approximately two hours) after semen collection, ensuring a cooling rate of approximately 0.10–0.15 °C/min.

### 2.5. Sperm Concentration and Kinetics

Upon arrival at the Laboratory of Biology and Technology of Animal Reproduction at the University of Basilicata, Potenza, all the semen samples were assessed for sperm concentration and kinetics using a Makler chamber (Sefi-Medical Instruments, Haifa, Israel) and computer-assisted sperm analysis (SCA 5.0 system, Microptic, Barcelona, Spain), as previously reported [17]. Briefly, following the evaluation of sperm concentration, each sample was diluted with INRA96^®^ to achieve a concentration of 30 × 10^6^ sperm mL^−1^ for SCA analysis. The samples were equilibrated for 2 min at 37 °C on a heated microscope stage. Spermatozoa with an average velocity of less than 10 μm s^−1^ were considered immotile. The sperm kinematic parameters assessed included the following: (i) the percentage of total motile spermatozoa (TM); (ii) the percentage of progressive spermatozoa (PM), defined as those with an average path velocity greater than 30 μm s^−1^ and a track straightness greater than 80%; (iii) curvilinear velocity (VCL, μm s^−1^) is the average velocity along the actual trajectory; (iv) straight-line velocity (VSL, μm s^−1^) is the velocity measured as a straight line between start and end points; and (v) average path velocity (VAP, μm s^−1^) is the velocity along a smoothed path of progression. For each sample, the tracks of at least one thousand spermatozoa were acquired.

### 2.6. Sperm Mitochondrial Membrane Potential (MMP)

Sperm samples (1 × 10^6^ spermatozoa) were stained with 1.5 μM JC-1, a potential-sensitive fluorescent dye that accumulates in mitochondria. Upon excitation at 488 nm, it emits at wavelengths of ~535 nm (J_0_A) and ~595 nm (J_0_B); the J_0_B/J_0_A ratio indicates the mitochondrial membrane potential (MMP) [18,19]. Samples were washed with phosphate-buffer saline supplemented with 0.1% polyvinyl alcohol (PBS-PVA) by centrifugation (300× *g* for 5 min), resuspended in 200 μL PBS-PVA, and incubated with JC-1 in the dark at RT for 30 min. After a second wash, the sperm pellets were resuspended in 700 μL PBS-PVA and incubated for another 30 min. Fluorescent spectra from 500 to 620 nm were recorded in duplicate using a spectrofluorometer (Cary Eclipse, Agilent Technologies, Rome, Italy).

### 2.7. Lipid Peroxidation (LPO) in Sperm Cells

Lipid peroxidation was measured using C11-BODIPY^581/591^ the fluorescence emission of which shifts from ~590 nm to ~520 nm upon oxidation [19]. Sperm samples (1 × 10^6^ cells) were incubated with 2 μM C11-BODIPY^581/591^ for 30 min at RT, centrifuged at 300× *g* for 5 min, resuspended in 700 μL of PBS-PVA, and incubated for another 30 min. Fluorescence was recorded using a spectrofluorometer, as described above. Lipid peroxidation was expressed as the ratio of the fluorescence peak at ~520 nm (C_0_A) to the sum of the peaks at ~520 nm and ~590 nm (C_0_A + C_0_B).

### 2.8. Intracellular Hydrogen Peroxide (H_2_O_2_) Content

The intracellular hydrogen peroxide (H_2_O_2_) content was assessed with 2′,7′-dichlorodihydrofluorescein diacetate (H_2_DCFDA) [20]. After a double wash with PBS-PVA by centrifugation (300× *g* for 5 min), sperm aliquots of 1 × 10^6^ sperm cells were stained with 10 μM H_2_DCFDA for 30 min in the dark at RT, washed, and then further incubated for 30 min in 700 μL PBS-PVA. After incubation, the samples were analyzed with a spectrofluorometer, as described above. The peak fluorescence intensity at ~525 nm following excitation at a wavelength of 488 nm is proportional to intracellular H_2_O_2_ levels, and is measured as arbitrary units (a.u.).

### 2.9. Intracellular Nitric Oxide (NO) Content

Intracellular nitric oxide (NO) was measured using 10 μM 4-amino-5-methylamino-2′,7′-difluorofluorescein diacetate (DAF-FM) [21]. Sperm samples (1 × 10^6^ cells) were stained for 30 min at RT in the dark, washed, and incubated in 700 μL PBS-PVA for another 30 min. Fluorescence was then measured (~525 nm emission, 488 nm excitation) with a spectrofluorometer, as described above. NO levels were expressed in arbitrary units (a.u.).

### 2.10. Sperm Freezing

The freezing extenders used in this trial were INRA Freeze^®^ (DLM Meazza, Lodi, Italy) [16] and HF-20 [15]. INRA Freeze^®^ is a patented commercial product with an undisclosed formulation, derived from INRA96. It contains electrolytes, buffers, sugars, cryoprotectants (glycerol), purified milk micellar proteins, egg yolk plasma, and antimicrobial agents (penicillin, gentamicin, amphotericin B). HF-20 is a semi-defined medium composed of 5 g glucose, 0.3 g lactose, 0.3 g raffinose, 0.15 g sodium citrate, 0.05 g sodium phosphate, 0.05 g potassium sodium tartrate, 25,000 IU penicillin, 0.08 g streptomycin, 3% glycerol, and ultrapure water to a final volume of 100 mL. To this base, 10% (*v*/*v*) fresh egg yolk (EY) was added, representing the only undefined component. To minimize biological variability, the EY was pooled prior to incorporation into the medium during preparation [17]. This freezing extender was stored at −30 °C in aliquots until it was ready to be used. After assessing sperm concentration, an adequate volume of diluted semen, assuring the packaging of seven straws for each freezing extender, was centrifuged at 400× *g* for 10 min. The sperm pellets were resuspended and the sperm was distributed in 3.5 mL of both INRA freeze and HF-20 to achieve a sperm concentration of 100 × 10^6^ sperm mL^−1^. The samples were placed in tubes and cooled gradually from +20 °C to +4 °C over 60 min, then moved to a +4 °C cold cabinet for straw packaging. The packaged doses were kept at +4 °C for 15 min before exposure to LN_2_ vapors [17].

### 2.11. Sperm Packaging, Freezing, and Thawing

Semen doses were filled into individually labeled 0.5 mL polyvinyl chloride straws (IMV-Technologies, L’Aigle, France) which had been pre-cooled to +4 °C. The straws were sealed with blue sealing powder (Poudre de bouchage bleue, IMV-Technologies, Cremona, Italy) and placed horizontally on a freezing rack (40-straw distribution block, IMV-Technologies). For the freezing process, the rack was placed in a polystyrene container filled with liquid nitrogen (LN_2_), with the straws held 4 cm above the LN_2_ surface for 10 min before being fully submerged for storage. One week after freezing, seven straws per extender per stallion were thawed—first for 10 s in air, and then for 30 s in a 37 °C water bath—immediately before semen analysis. An initial centrifugation step was performed to reduce the semen volume. Each sample was then layered onto a 60/40 discontinuous Percoll gradient and centrifuged at 800× *g* for 10 min to eliminate egg yolk residues that could interfere with subsequent sperm analyses. The resulting pellet was resuspended in a small volume of PBS-PVA and divided into two aliquots. One aliquot was diluted in INRA96^®^ and analyzed microscopically for sperm kinetics. The second aliquot was washed with PBS-PVA and used to evaluate mitochondrial activity, lipid peroxidation (LPO), and the levels of hydrogen peroxide (H_2_O_2_) and nitric oxide (NO).

### 2.12. Statistical Analysis

The data were analyzed with ANOVA for repeated measurements (independent variables: stallion, extender, and age) using Systat (Systat 11.0 release, Systat Software, Inc., San Jose, CA, USA). Multivariate linear regression analysis was conducted to compare sperm function in refrigerated and frozen/thawed samples; the regression model included stallion, extender, and age effects. Before the analyses were carried out, percentage values were transformed in arcsine. Normal data distribution and homogeneity of variance were assessed using the Shapiro–Wilk test and Levene’s test, respectively. Pairwise comparisons of the means were performed with Tukey’s test. A threshold of *p* < 0.05 was used as the minimum level of statistical significance. The data are presented as the mean ± standard deviation (SD). The linear regression procedure (Systat 11.0 release) was applied to calculate the coefficients of correlation (R). Principal component analysis was performed using the open-source software JASP 0.18.3 (University of Amsterdam, Amsterdam, The Netherlands).

Repeatability (*r*) was estimated by partitioning the total phenotypic variance of semen traits among stallions into additive genetic, permanent, and temporary environmental components [22]. The permanent environmental variance represents the proportion of variance that is attributable to environmental factors that exert consistent and long-term effects on individual stallions. The temporary environmental variance represents transitional effects that vary from one period to the next. To extract the sources of variance among stallions associated with housing conditions, season and nutrition, and the date of samplings, the order of sampling, a progressive number from the 1st to the 15th sampling session was included in the model. The residual variance reflects the variance observed within stallions. Hence, repeatability for semen traits was estimated as *r* = σ^2^_a_ + σ^2^_p_/(σ^2^_a_ + σ^2^_p_ + σ^2^_t_ + σ^2^_e_), where

σ^2^_a_ = additive genetic variance among stallions;

σ^2^_p_ = permanent environmental variance among stallions;

σ^2^_t_ = temporary environmental variance among stallions;

σ^2^_e_ = residual variance.

## 3. Results

The mean results of the four repeated weekly semen collections from the 11 evaluated Salernitano stallions are presented in Table 1. Significant individual variability was observed in the volume of gel-free semen, as well as in the total motility, VCL, and lipoperoxidation of the freshly collected sperm. The repeatability of these parameters fell within a range that can be described as low to slightly below moderate. In contrast, all of the other assessed parameters related to the kinematic, bioenergetic, and oxidative/nitrosative stress-related properties of the spermatozoa showed no significant variability among stallions. Stallion age did not show statistically significant effects on any of the parameters considered in the analysis of variance, except for semen volume (*p* = 0.004), total motility (*p* < 0.001), and lipid peroxidation level (*p* = 0.007). However, in the regression analysis used to assess correlations, statistically significant results emerged for semen volume, which was positively correlated with age (R = +0.669, *p* = 0.001), and for total and progressive motility, which were negatively correlated with age (TM: R = −0.707, *p* < 0.001; PM: R = −0.565, *p* = 0.023) (Table S1).

Table 2 shows the physiological parameters related to sperm kinetics, bioenergetics, and oxidative/nitrosative stress markers measured after the thawing sperm samples frozen with the two different extenders, HF-20 and INRA Freeze. Overall, no statistically significant differences were observed between the extenders across most parameters, except for higher H_2_O_2_ (*p* = 0.05) and NO (*p* < 0.01) levels in spermatozoa frozen with INRA Freeze. However, significant variability among stallions and low-to-moderate repeatability were detected for all kinematic parameters, mitochondrial membrane potential, lipoperoxidation, and NO content in sperm samples frozen with INRA Freeze. Sperm samples frozen with HF-20 exhibited significant differences among stallions and low repeatability only in relation to progressive motility and H_2_O_2_ content. In principal component analysis, component 1, which captures the greatest variance in the data and combines the variables with the highest dispersion, contains most of the kinematic data (VCL, VSL, VAP, PM) and age. Among these variables, however, the predominant role of the kinematic variables related to sperm velocity (VCL, VSL, and VAP) is clearly evident. On the contrary, the effect of the two freezing extenders appears to be negligible. Comparison of the results obtained before freezing (Table 1) and after freezing (Table 2) revealed a significant reduction (*p* < 0.001) in all sperm kinematic parameters and in mitochondrial membrane potential (MMP) for both extenders. Following cryopreservation, the levels of lipid peroxidation (LPO), H_2_O_2_, and NO increased significantly (*p* < 0.001) in spermatozoa frozen with INRA Freeze. In contrast, spermatozoa frozen with HF-20 showed a significant increase in LPO levels (*p* = 0.04), while their H_2_O_2_ levels increased and their NO levels decreased compared to the fresh samples; however, these latter two changes were not statistically significant. Significant effects of stallion age on sperm parameters were observed for LPO and H_2_O_2_ content in spermatozoa cryopreserved with HF-20, whereas in spermatozoa frozen with INRA Freeze, age effects were observed for sperm kinematic parameters, LPO, and NO content. In addition, numerous significant correlations were observed between age and each of the parameters measured in spermatozoa frozen with both extenders (Table S1).

Table 3 presents the analysis of the ratios between post-thaw and pre-freezing values of the evaluated parameters, which are used as indices of sperm freezability encompassing all measured aspects of sperm physiology. High individual variability and low-to-moderate repeatability were observed for all kinematic parameters, except progressive motility (PM), in sperm frozen with INRA Freeze. In contrast, sperm frozen with HF-20 showed no significant differences in their freezability indices among stallions. Neither intracellular H_2_O_2_ levels nor NO levels showed significant variation among stallions, and both exhibited very low repeatability. Stallion age did not influence freezability based on the selected sperm parameters, except for VCL in spermatozoa frozen with INRA Freeze. However, significant correlations between age and freezability were observed in sperm frozen with both HF-20 and INRA Freeze^®^ (Table S2). When stallions were grouped into two age categories (≤10 and >10 years old), significant differences were detected in semen volume and sperm concentration, as well as in the total motility and NO content in fresh semen (Table S3). Significant age-related differences were also observed in VCL and H_2_O_2_ content in semen frozen with HF-20, and in kinematic parameters (VCL, VSL, and VAP) in spermatozoa frozen with INRA Freeze^®^ (Table S4).

Potential associations between sperm physiological parameters measured pre-freezing the semen and their corresponding freezability indices, calculated as the ratio of post-thaw to pre-freezing values, are presented in Table 4. This analysis provides an estimate of the predictive value of the sperm’s pre-freezing parameters in relation to their cryotolerance. For both freezing extenders tested, the results clearly show that total and progressive motility are not reliable predictors of freezability. In contrast, significant correlations were observed for sperm kinematic parameters (VCL, VSL, and VAP), as well as for mitochondrial activity and lipoperoxidation. Although the differences were modest, significant correlations for H_2_O_2_ and NO content were found only in spermatozoa cryopreserved with HF-20.

Finally, all the physiological parameters assessed in Salernitano stallion sperm were analyzed for pairwise correlations, and the corresponding correlation coefficients, along with their statistical significance, are presented in Table 5. All the sperm kinematic parameters exhibited strong and statistically significant correlations with one another. Similarly, mitochondrial membrane potential (MMP) showed strong and significant correlations with all the sperm kinematic parameters. Lipoperoxidation (LPO) was significantly negatively correlated with most of the sperm kinematic parameters, except for total motility. Additionally, LPO showed a strong positive correlation with nitric oxide (NO) levels. In contrast, H_2_O_2_ and NO levels did not display significant correlations with most of the other sperm functional parameters; however, a strong and significant positive correlation was observed between them.

## 4. Discussion

The low semen freezability observed in some stallions highlights the need for specialized freezing extenders to enhance the cryotolerance of sperm cells. This approach could be successful if cryotolerance is primarily influenced by temporary and reversible environmental factors, rather than by genetic traits or permanent environmental conditions. Our analysis of the repeatability of selected sperm quality parameters in fresh and frozen–thawed spermatozoa, as well as of freezability, yielded informative results. The magnitude of variation among stallions, together with the degree of variation among repeated measurements within the same stallion, determines the corresponding value of trait repeatability [23]. The low-to-moderate repeatability observed in most of the selected sperm physiological parameters indicates, on the one hand, the limited capacity of individual stallions to maintain consistent values of a given parameter over time, and on the other hand, the substantial influence of transient environmental variance on each parameter. This suggests that these traits are not strongly constrained by genetic or permanent environmental factors, and could, therefore, be modulated through targeted interventions, offering considerable scope for the use of freezing extenders tailored to individual stallion requirements.

In the present study, the overall reduction observed in all sperm kinematic parameters and mitochondrial activity in spermatozoa frozen with both extenders compared to fresh semen, accompanied by an increase in oxidative and nitrosative stress markers, was an expected outcome, and is consistent with findings reported in previous studies [24]. Similarly, the absence of significant differences in freezing efficiency and sperm function between sperm frozen with the two extenders is also consistent with previous research [17]. However, the results obtained from repeated semen sampling in the same animals revealed novel and noteworthy findings. Through comparing the post- and pre-freezing values for each parameter, a panel of sperm freezability assessments was obtained. The analyses conducted allowed us to verify the degree of variability in each parameter among the stallions, as well as its repeatability. In particular, semen volume varied considerably among stallions, a difference that may be attributable to the large age range of the animals, as shown in Table S3, and which is consistent with the findings of Dowsett and Knott [25]. The low repeatability (0.32) of this characteristic is consistent with the findings of a previous study on Lusitano stallions [12], which reported a repeatability value of 0.35. However, compared to that study, we observed lower repeatability values for sperm concentration (0.15 vs. 0.38) and the total number of spermatozoa per ejaculate (0.08 vs. 0.41).

Total motility (TM) and progressive motility (PM) are among the most commonly used sperm parameters due to their ease of measurement and perceived reliability [26]. However, their usefulness as indicators of fertility and sperm freezability is uncertain. In fresh semen, only weak correlations were observed between fertility and the percentages of total motile (R = 0.40) and progressively motile (R = 0.46) spermatozoa [27]. Similarly, in frozen semen, the predictive value of these assessments remains limited; however, it may be enhanced when combined with additional evaluations, such as sperm morphology and viability, membrane integrity, DNA fragmentation, or novel biomarkers like proAKAP4 [28,29]. In our study, in fresh semen, TM exhibited significant individual variability, whereas PM did not. Spermatozoa frozen with both extenders showed significant variability in PM among stallions, together with low repeatability, whereas TM showed significant individual variation and low repeatability only in spermatozoa frozen with INRA Freeze. Analysis of the freezability index based on these parameters revealed a significant stallion effect, together with low repeatability, only for TM in samples frozen with INRA Freeze. Moreover, the correlations between the pre-freezing values of TM and PM and their respective freezability indices were low and not statistically significant. As such, these kinematic sperm parameters have limited predictive value for assessing the individual freezability potential of stallion semen. This finding aligns with observations in heavy draft stallions whose semen were evaluated across reproductive seasons [5]. In that study, no significant differences were found in sperm kinematic parameters between in-season and out-of-season collections; however, significant season-related differences occurred only in the freezability index related to sperm viability.

Interesting findings were also obtained for additional sperm kinematic indices associated with velocity, such as VCL, VSL, and VAP. These sperm parameters are obtainable through computerized tracking of individual spermatozoa over very short periods of time. This method of sperm evaluation is accurate and is no longer subjective, and as such, it is widely used for sperm analysis in humans, as well as in many animal species [30], including equines [31]. In stallions, as in other animal species, this methodology has progressively improved the repeatability of accuracy, but its reproducibility between laboratories remains questionable due to differences in settings [32]. In the present study, these parameters were strongly correlated with each other and also showed significant associations with other kinematic parameters (TM and PM), mitochondrial activity, and lipoperoxidation. In fresh sperm, however, only VCL showed statistically significant differences between stallions, and even then, with low repeatability. In samples frozen using INRA Freeze, however, all three kinematic parameters (VCL, VSL, and VAP) exhibited significant inter-stallion differences with moderate repeatability. This pattern was not observed in samples frozen with HF-20. When freezability indices based on these parameters were analyzed, sperm frozen with INRA Freeze again demonstrated significant differences with low-to-moderate repeatability. Moreover, significant correlations were found between the pre-freezing values of these parameters and the freezability index for both extenders. The ability of INRA Freeze to yield more homogeneous and repeatable outcomes in certain stallions suggests that sperm freezability depends on both individual stallion characteristics and the choice of freezing extender. While INRA Freeze and HF-20 performed similarly in preserving kinematic parameters, mitochondrial activity, and lipoperoxidation levels, they differed in their effects on sperm H_2_O_2_ and NO content. The freezability index indicated greater variability and repeatability in the parameters of sperm frozen with INRA Freeze, implying stronger discrimination between stallions, whereas HF-20 produced more uniform results across individuals. These findings align with the results of a previous study [17], which reported no overall statistical differences among extenders, but highlighted superior outcomes in specific extender–stallion combinations. Also in principal component analysis, these kinematic parameters exhibit the highest variance and greatest data dispersion, while the variance attributable to the freezing extender remains comparatively low. To date, the predictive value of sperm kinematic parameters for field fertility has shown inconsistent outcomes. During the breeding season, stallions classified as “fertile” exhibited significantly higher VAP and VCL values compared to sub-fertile stallions; however, these differences were not observed outside the breeding season [33]. Furthermore, a review of frozen stallion semen revealed only weak correlations between sperm kinematic parameters and pregnancy rates [34]. In contrast, sperm kinematic parameters have yielded more consistent results in other species, such as cattle. A large-scale insemination study on bull semen identified VAP as the most reliable kinematic parameter for predicting fertility [35]. Based on the findings of the present study and supporting evidence from other species, the predictive value of these kinematic parameters in stallion spermatozoa may need to be reconsidered.

Mitochondrial membrane potential (MMP) is widely used as an indicator of sperm bioenergetic activity [36], as it dynamically reflects the cell’s metabolic state. Rather than capturing a progressive change, MMP provides a snapshot of the metabolic status at the precise moment of measurement. However, this feature can lead to inaccurate or inconsistent results if the evaluation is conducted under suboptimal conditions or if analysis times are prolonged. As a result, reproducibility across laboratories may vary depending on the specific protocols and settings used [37]. In the present study, MMP was significantly correlated with all sperm kinematic parameters, consistent with previous findings in cattle [18,20,38], but only partially in agreement with studies on warmblood stallions, where MMP correlated significantly only with PM, superoxide anion content, and ATP content [39]. MMP did not differ significantly among stallions in fresh spermatozoa; however, inter-stallion differences emerged in frozen–thawed samples. The absence of variation in fresh semen cannot be attributed to age effects (Tables S1 and S3), whereas the differences in frozen samples may reflect the distinct freezing responses of stallions with low versus high freezability [40]. Nevertheless, when evaluated as an indicator of freezability, MMP did not vary significantly among stallions, in agreement with earlier studies comparing pre- and post-freezing results in stallion spermatozoa [17], which reported no significant differences between individuals or between freezing extenders, apart from the effects of the freezing itself. Nevertheless, the correlation coefficient between MMP in fresh spermatozoa and the corresponding freezability index was high and statistically significant for both freezing extenders. This suggests that MMP levels in fresh semen have good predictive value for assessing sperm freezability, at least with respect to this specific sperm function.

Owing to their high content of polyunsaturated fatty acids, which are essential for maintaining membrane fluidity, sperm plasma membranes are particularly susceptible to reactive oxygen and nitrogen species (ROS and RNS), which can trigger lipid peroxidation (LPO) [41,42]. In particular, certain ROS, such as hydrogen peroxide, target these fatty acids, generating reactive peroxyl and alkoxyl radicals. In order to stabilize, these radicals extract hydrogen atoms from neighboring lipids, producing lipid acids or alcohols and generating new carbon-centered radicals. These, in turn, react with oxygen to form additional lipid peroxides, initiating a chain reaction that exacerbates membrane damage [43,44]. LPO poses a serious threat to membrane fluidity and can significantly impair sperm motility [20,45]. The sperm cell’s minimal cytoplasmic volume and limited antioxidant defenses, particularly when seminal plasma is removed or diluted during equine sperm cryopreservation [46], further contribute to its high susceptibility to oxidative damage [47,48]. In the present study, hydrogen peroxide (H_2_O_2_) and nitric oxide (NO) were measured as representatives of ROS and RNS, respectively. LPO was assessed using a fluorescent probe, providing a snapshot of membrane damage that has already occurred, regardless of the current levels of ROS or RNS [49,50]. While the presence of these radicals can signal both oxidative stress risk and sperm metabolic activity, their concentrations can be misleading [51]. A self-reinforcing cycle may develop in which elevated sperm metabolism increases free radical production, ultimately impairing the metabolism itself. Without sufficient antioxidant protection, this can lead to a progressive decline in free radical levels, not due to recovery, but as a result of metabolic collapse. Consequently, high LPO levels may coexist with reduced ROS concentrations, indicating prior oxidative damage. In this study, the LPO levels in fresh semen varied significantly among stallions, while intracellular H_2_O_2_ and NO concentrations remained relatively stable. Although LPO levels increased significantly after cryopreservation with both freezing extenders, significant inter-stallion differences and moderate repeatability were found in spermatozoa frozen with INRA Freeze. However, no significant differences between stallions were detected with either freezing extender when the corresponding freezability indices were considered. In contrast, spermatozoa cryopreserved with HF-20 showed a significant correlation between pre-freezing H_2_O_2_ and NO levels and their respective freezability values, an outcome not observed with INRA Freeze.

Stallion age, a permanent environmental factor that can influence sperm quality and freezability, affected semen quality in both fresh samples and samples frozen with both extenders, although its impact on freezability was modest. This finding is consistent with a previous study on different equine breeds subjected to semen freezing [52], which found that stallions over 9 years of age were significantly more likely to be excluded from insemination programs due to their sperm exhibiting a post-thaw progressive motility value below 35%. Moreover, in that study, the importance of age as an independent variable for predicting the percentage of acceptable ejaculates, defined as 100% for total motility, declined to 19%.

## 5. Conclusions

Repeated semen collections from stallions, cryopreserved using two extenders, INRA Freeze and HF-20, were analyzed over time to assess the repeatability of sperm quality parameters, including kinetics, bioenergetics, and oxidative/nitrosative stress markers. Both extenders preserved sperm efficiently; however, INRA Freeze demonstrated a greater capacity to discriminate between individuals, with low-to-moderate repeatability across sperm physiological parameters, compared with HF-20. The low-to-moderate repeatability observed in specific sperm physiological traits highlights the critical role of the freezing extender in optimizing cryopreservation conditions for distinct sperm subpopulations, and supports the development of individualized strategies to enhance the resilience of equine sperm to cryo-induced stress.

## Figures and Tables

**Table 1:** Mean values (±SD), 75% confidence interval, inter-stallion variability, and repeatability of selected parameters in fresh semen, including gel-free semen volume, sperm concentration, total sperm count, and physiological characteristics, such as sperm kinetics, bioenergetics, and oxidative/nitrosative stress markers.

		Mean ± SD	75% Confidence Interval	Inter-Stallion Variability (*p*=)	Repeatability (*r*)
Gel-free volume	mL	43.9 ± 25.0	39.6 ÷ 48.3	0.015 *	0.32
Sperm concentration	×10^6^	297 ± 164	269 ÷ 325	0.142	0.15
Spermatozoa per ejaculate	×10^9^	12.3 ± 10.0	10.6 ÷ 14.1	0.263	0.08
TM	%	83.0 ± 12.9	80.7 ÷ 85.3	0.007 **	0.38
PM	%	32.9 ± 10.9	31.0 ÷ 34.9	0.122	0.17
VCL	µm s^−1^	91.8 ± 16.4	88.9 ÷ 94.8	0.019 *	0.32
VSL	µm s^−1^	36.5 ± 6.9	35.2 ÷ 37.7	0.250	0.01
VAP	µm s^−1^	50.0 ± 9.5	48.3 ÷ 51.7	0.434	0.01
MMP	J_0_B/J_0_A	13.7 ± 9.2	12.3 ÷ 15.2	0.291	0.08
LPO	C_0_A/(C_0_A + C_0_B)	11.9 ± 4.1	11.2 ÷ 12.6	0.020 *	0.36
H_2_O_2_ content	a.u.	3.0 ± 1.4	2.7 ÷ 3.2	0.073	0.26
NO content	a.u.	2.6 ± 1.2	2.4 ÷ 2.8	0.104	0.25

Total motility (TM), progressive motility (PM), curvilinear velocity (VCL), straight-line velocity (VSL), average path velocity (VAP), mitochondrial membrane potential (MMP), lipid peroxidation (LPO), hydrogen peroxide (H_2_O_2_), and nitric oxide (NO). MMP was calculated as the ratio of the second (~595 nm) to the first (~535 nm) fluorescence intensity peak (J_0_B/J_0_A). LPO was calculated as the ratio of the first (~520 nm) fluorescence intensity peak to the sum of the first (~520 nm) and second (~590 nm) fluorescence intensity peaks, expressed as C_0_A/(C_0_A + C_0_B). a.u.: arbitrary units of fluorescence. * (*p* ≤ 0.05), ** (*p* ≤ 0.01).

**Table 2:** Mean values (±SD), 75% confidence interval, inter-stallion variability, and repeatability of selected parameters, including sperm kinetics, bioenergetics, and oxidative/nitrosative stress markers, in stallion sperm samples cryopreserved with two freezing extenders (HF-20 and INRA Freeze^®^).

		Freezing Extender	Mean ± SD	75% Confidence Interval	Inter-Stallion Variability (*p*=)	Repeatability (*r*)
TM	%	HF-20	41.9 ± 20.3	38.2 ÷ 45.5	0.161	0.14
		INRA Freeze	43.9 ± 20.0	40.3 ÷ 47.6	0.030 *	0.30
PM	%	HF-20	11.3 ± 11.2	9.3 ÷ 13.4	0.049 *	0.25
		INRA Freeze	9.7 ± 8.1	8.2 ÷ 11.2	0.015 *	0.35
VCL	µm s^−1^	HF-20	34.6 ± 15.4	31.8 ÷ 37.4	0.209	0.11
		INRA Freeze	36.0 ± 13.2	33.6 ÷ 38.4	0.001 ***	0.61
VSL	µm s^−1^	HF-20	16.0 ± 7.9	14.6 ÷ 17.4	0.403	0.02
		INRA Freeze	15.7 ± 6.4	14.6 ÷ 16.9	0.001 ***	0.50
VAP	µm s^−1^	HF-20	19.9 ± 8.6	18.3 ÷ 21.4	0.379	0.03
		INRA Freeze	20.1 ± 7.3	18.8 ÷ 21.5	0.001 ***	0.55
MMP	F_0_B/F_0_A	HF-20	7.3 ± 4.6	6.5 ÷ 8.0	0.051	0.17
		INRA Freeze	7.7 ± 8.2	6.4 ÷ 9.0	0.007 **	0.29
LPO	F_0_A/(F_0_A + F_0_B)	HF-20	13.9 ± 5.8	13.0 ÷ 14.8	0.067	0.15
		INRA Freeze	15.5 ± 6.0	14.6 ÷ 16.5	0.001 ***	0.47
H_2_O_2_ content	a.u.	HF-20	2.5 ± 2.6 **a**	2.1 ÷ 2.9	0.011 *	0.25
		INRA Freeze	3.6 ± 2.3 **b**	3.2 ÷ 3.9	0.744	0.01
NO content	a.u	HF-20	2.2 ± 1.4 **A**	2.0 ÷ 2.4	0.051	0.17
		INRA Freeze	4.2 ± 2.8 **B**	3.7 ÷ 4.6	0.048 *	0.20

Total motility (TM), progressive motility (PM), curvilinear velocity (VCL), straight-line velocity (VSL), average path velocity (VAP), mitochondrial membrane potential (MMP), lipid peroxidation (LPO), hydrogen peroxide (H_2_O_2_), and nitric oxide (NO). MMP was calculated as the ratio of the second (~595 nm) to the first (~535 nm) fluorescence intensity peak (J_0_B/J_0_A). LPO was calculated as the ratio of the first (~520 nm) fluorescence intensity peak to the sum of the first (~520 nm) and second (~590 nm) fluorescence intensity peaks, expressed as C_0_A/(C_0_A + C_0_B). a.u.: arbitrary units of fluorescence. Between extenders: a, b (*p* ≤ 0.05), A, B (*p* ≤ 0.01). Among stallions: * (*p* ≤ 0.05), ** (*p* ≤ 0.01), *** (*p* ≤ 0.001).

**Table 3:** Mean values (±SD), 75% confidence interval, inter-stallion variability, and repeatability of freezability, assessed as the ratio of post-thaw to fresh values, of selected sperm physiological parameters including sperm kinetics, bioenergetics, and oxidative/nitrosative stress markers, in stallion sperm samples cryopreserved with two different freezing extenders (HF-20 and INRA Freeze^®^).

		Freezing Extender	Mean ± SD	75% Confidence Interval	Inter-Stallion Variability (*p*=)	Repeatability (*r*)
TM	%	HF-20	50.2 ± 23.0	46.1 ÷ 54.4	0.453	0.06
	%	INRA Freeze	52.5 ± 21.9	48.5 ÷ 56.5	0.021 *	0.33
PM	%	HF-20	33.7 ± 31.9	27.9 ÷ 39.4	0.230	0.13
	%	INRA Freeze	29.1 ± 21.6	25.1 ÷ 33.0	0.131	0.17
VCL	%	HF-20	38.9 ± 21.4	35.0 ÷ 42.7	0.293	0.19
	%	INRA Freeze	40.3 ± 17.4	37.1 ÷ 43.5	0.001 ***	0.56
VSL	%	HF-20	45.7 ± 28.0	40.6 ÷ 50.7	0.521	0.01
	%	INRA Freeze	44.6 ± 20.9	40.8 ÷ 48.4	0.026 *	0.31
VAP	%	HF-20	41.5 ± 22.9	37.3 ÷ 45.6	0.464	0.01
	%	INRA Freeze	41.6 ± 17.7	38.4 ÷ 44.9	0.010 **	0.38
MMP	%	HF-20	80.6 ± 69.8	65.4 ÷ 95.9	0.552	0.01
	%	INRA Freeze	76.9 ± 64.1	63.0 ÷ 91.2	0.339	0.04
LPO	%	HF-20	125.7 ± 59.8	112.7 ÷ 138.8	0.600	0.01
	%	INRA Freeze	138.8 ± 63.7	124.6 ÷ 152.9	0.364	0.06
H_2_O_2_ content	%	HF-20	107.3 ± 105.4	82.4 ÷ 132.1	0.377	0.05
	%	INRA Freeze	150.1 ± 116.6	122.0 ÷ 178.2	0.929	0.01
NO content	%	HF-20	88.4 ± 62.3	72.7 ÷ 104.2	0.460	0.01
	%	INRA Freeze	159.4 ± 119.1	128.6 ÷ 190.2	0.865	0.01

Total motility (TM), progressive motility (PM), curvilinear velocity (VCL), straight-line velocity (VSL), and average path velocity (VAP), mitochondrial membrane potential (MMP), lipid peroxidation (LPO), hydrogen peroxide (H_2_O_2_), and nitric oxide (NO). * (*p* ≤ 0.05), ** (*p* ≤ 0.01), *** (*p* ≤ 0.001).

**Table 4:** Pearson correlation coefficients (R) between fresh sperm parameters and their corresponding freezability (post-/pre-freezing ratio), based on sperm kinetics, bioenergetics, and oxidative/nitrosative stress markers in stallion sperm samples cryopreserved with two different freezing extenders (HF-20 and INRA Freeze^®^).

	HF-20 R	INRA Freeze R
TM	+0.050	+0.146
PM	+0.076	+0.082
VCL	−0.310 *	−0.396 **
VSL	−0.356 *	−0.378 **
VAP	−0.399 **	−0.424 **
MMP	−0.542 **	−0.348 *
LPO	−0.399 *	−0.476 **
H_2_O_2_ content	−0.381 *	−0.376
NO content	−0.465 *	−0.322

Total motility (TM), progressive motility (PM), curvilinear velocity (VCL), straight-line velocity (VSL), average path velocity (VAP), mitochondrial membrane potential (MMP), lipid peroxidation (LPO), hydrogen peroxide (H_2_O_2_), and nitric oxide (NO). * (*p* ≤ 0.05), ** (*p* ≤ 0.01).

**Table 5:** Pairwise correlation coefficients (R) between sperm total motility (TM) progressive motility (PM), curvilinear velocity (VCL), straight-line velocity (VSL), average path velocity (VAP), mitochondrial membrane potential (MMP), lipid peroxidation (LPO), hydrogen peroxide (H_2_O_2_) content, and nitric oxide (NO) content, evaluated across all sperm samples (both fresh and frozen–thawed with both extenders).

	PM	VCL	VSL	VAP	MMP	LPO	H_2_O_2_	NO
TM	+0.841 ***	+0.696 ***	+0.674 ***	+0.699 ***	+0.325 **	−0.140	+0.116	+0.088
PM		+0.777 ***	+0.828 ***	+0.783 ***	+0.218 **	−0.180 *	+0.116	+0.156
VCL			+0.956 ***	+0.980 ***	+0.274 **	−0.236 *	+0.041	−0.149
VSL				+0.984 ***	+0.239 *	−0.228 *	+0.043	−0.159
VAP					+0.271 **	−0.227 *	+0.036	−0.153
MMP						−0.065	+0.041	−0.120
LPO							+0.145	+0.468 ***
H_2_O_2_								+0.543 ***

* (*p* ≤ 0.05), ** (*p* ≤ 0.01), *** (*p* ≤ 0.001).

## Data Availability

The original contributions presented in this study are included in the article/Supplementary Material. Further inquiries can be directed to the corresponding author.

## Supplementary Materials

The following supporting information can be downloaded at https://www.mdpi.com/article/10.3390/ani15192805/s1. Table S1: Effect of stallion age on semen and sperm parameters in both fresh and frozen–thawed samples (using two extenders: HF-20 and INRA Freeze), with corresponding Pearson correlation coefficients; Table S2: Effect of stallion age on sperm freezability, expressed as the ratio of thawed to fresh values, with corresponding Pearson correlation coefficients; Table S3: Semen and fresh sperm parameters in stallions grouped by age; Table S4: Post-thaw sperm parameters in stallions grouped by age; Figure S1: Typical emission spectral diagrams recorded for stallion sperm loaded with different fluorochromes and their respective positive controls; Figure S2: Principal component analysis (PCA) outcomes.

## References

[1] Al-Kass Z., Morrell J.M. (2024). Freezing Stallion Semen-What Do We Need to Focus on for the Future?. Vet. Sci..

[2] Ferreira H.N., Ferreira-Silva J.C., Rocha J.M., Farras M.C., Calixto M., Moura M.T., Alvarenga M.A., Oliveira A.L. (2018). Variable Inter-assay Estimation of Sperm DNA Fragmentation In Stallions Classified as Good and Bad Semen Freezers. Cryo Lett..

[3] Hoffmann N., Oldenhof H., Morandini C., Rohn K., Sieme H. (2011). Optimal concentrations of cryoprotective agents for semen from stallions that are classified ‘good’ or ‘poor’ for freezing. Anim. Reprod. Sci..

[4] Samper J.C., Morris C.A. (1998). Current methods for stallion semen cryopreservation: A survey. Theriogenology.

[5] Ebel F., Vallejos A., Gajardo G., Ulloa O., Clavel E., Rodríguez-Gil J.E., Ramírez-Reveco A. (2020). Semen quality and freezability analysis during breeding and non-breeding seasons in heavy draft stallions in southern Chile. Andrologia.

[6] Love C.C., Thompson J.A., Brinsko S.P., Rigby S.L., Blanchard T.L., Lowry V.K., Varner D.D. (2003). Relationship between stallion sperm motility and viability as detected by two fluorescence staining techniques using flow cytometry. Theriogenology.

[7] Giannoccaro A., Lacalandra G.M., Filannino A., Pizzi F., Nicassio N., Dell’Aquila M.E., Minervini F. (2010). Assessment of viability, chromatin structure stability, mitochondrial function and motility of stallion fresh sperm by using objective methodologies. J. Cell Anim. Biol..

[8] Moraes C.R., Runcan E.E., Blawut B., Coutinho da Silva M.A. (2019). Technical Note: The use of iSperm technology for on-farm measurement of equine sperm motility and concentration. Transl. Anim. Sci..

[9] Janett F., Thun R., Niederer K., Burger D., Hässig M. (2003). Seasonal changes in semen quality and freezability in the Warmblood stallion. Theriogenology.

[10] Dohm M. (2002). Repeatability estimates do not always set an upper limit to heritability. Funct. Ecol..

[11] Harper D.G.C. (1994). Some comments on the repeatability of measurements. Ring. Migr..

[12] Gonçalves A.R., Telo da Gama L., Antunes L., Guimarães H., Bliebernicht M., Duarte J.C., Cosinha C., Duarte Rego B., Ferro da Costa P., Guimarães T. (2023). Impact of inbreeding and genetic parameter estimates for seminal traits in Lusitano horses. Theriogenology.

[13] Gottschalk M., Sieme H., Martinsson G., Distl O. (2016). Heritability of semen traits in German Warmblood stallions. Anim. Reprod. Sci..

[14] Greiser T., Sieme H., Martinsson G., Distl O. (2019). Genetic parameters and estimated breeding values for traits of raw and frozen-thawed semen in German Warmblood stallions. Anim. Reprod. Sci..

[15] Nishikawa Y. (1975). Studies on the preservation of raw and frozen horse semen. J. Reprod. Fertil. Suppl..

[16] Pillet E., Labbe C., Batellier F., Duchamp G., Beaumal V., Anton M., Desherces S., Schmitt E., Magistrini M. (2012). Liposomes as an alternative to egg yolk in stallion freezing extender. Theriogenology.

[17] Boni R., Ruggiero R., Di Palma T., Ferrara M.A., Preziosi G., Cecchini Gualandi S. (2024). Stallion Sperm Freezing with Different Extenders: Role of Antioxidant Activity and Nitric Oxide Production. Animals.

[18] Boni R., Gallo A., Cecchini S. (2017). Kinetic activity, membrane mitochondrial potential, lipid peroxidation, intracellular pH and calcium of frozen/thawed bovine spermatozoa treated with metabolic enhancers. Andrology.

[19] Di Palma T., Cecchini S., Macchia G., Pasolini M.P., Cocchia N., Boni R. (2020). Kinematic, bioenergetic and oxidative evaluations of donkey sperm preserved at +4 °C. Zygote.

[20] Gallo A., Esposito M.C., Tosti E., Boni R. (2021). Sperm Motility, Oxidative Status, and Mitochondrial Activity: Exploring Correlation in Different Species. Antioxidants.

[21] Chiu P.C.N., Liao S., Lam K.K.W., Tang F., Ho J.C.M., Ho P.C., O W.S., Yao Y.Q., Yeung W.S.B. (2010). Adrenomedullin regulates sperm motility and oviductal ciliary beat via cyclic adenosine 5′-monophosphate/protein kinase A and nitric oxide. Endocrinology.

[22] Boni R., Roelofsen M.W., Pieterse M., Kogut J., Kruip T.A. (1997). Follicular dynamics, repeatability and predictability of follicular recruitment in cows undergoing repeated follicular puncture. Theriogenology.

[23] Wolak M.E., Fairbairn D.J., Paulsen Y.R. (2012). Guidelines for estimating repeatability. Methods Ecol. Evol..

[24] Sielhorst J., Hagen C., Behrendt D., Schuette B., Burger D., Martinsson G., Sieme H. (2016). Effect of Multiple Freezing of Stallion Semen on Sperm Quality and Fertility. J. Equine Vet. Sci..

[25] Dowsett K.F., Knott L.M. (1996). The influence of age and breed on stallion semen. Theriogenology.

[26] Love C.C. (2018). Sperm quality assays: How good are they? The horse perspective. Anim. Reprod. Sci..

[27] Jasko D., Little T., Lein D., Foote R. (1992). Comparison of spermatozoal movement and semen characteristics with fertility in stallions: 64 cases (1987–1988). J. Am. Vet. Med. Assoc..

[28] Blommaert D., Sergeant N., Delehedde M., Donnay I., Lejeune J.P., Franck T., Serteyn D. (2021). First results about ProAKAP4 concentration in stallion semen after cryopreservation in two different freezing media. Cryobiology.

[29] Johannisson A., Morrell J.M., Ntallaris T. (2024). A combination of biomarkers for predicting stallion sperm fertility. Vet. Res. Commun..

[30] Mortimer S.T., van der Horst G., Mortimer D. (2015). The future of computer-aided sperm analysis. Asian J. Androl..

[31] Jasko D.J., Lein D.H., Foote R.H. (1991). The repeatability and effect of season on seminal characteristics and computer-aided sperm analysis in the stallion. Theriogenology.

[32] Egyptien S., Deleuze S., Ledeck J., Ponthier J. (2023). Sperm Quality Assessment in Stallions: How to Choose Relevant Assays to Answer Clinical Questions. Animals.

[33] Suliman Y., Becker F., Tuchscherer A., Wimmers K. (2020). Seasonal variations in quantitative and qualitative sperm characteristics in fertile and subfertile stallions. Arch. Anim. Breed..

[34] Katila T. (2001). In Vitro Evaluation of Frozen-Thawed Stallion Semen: A Review. Acta Vet. Scand..

[35] Nagy Á., Polichronopoulos T., Gáspárdy A., Solti L., Cseh S. (2015). Correlation between bull fertility and sperm cell velocity parameters generated by computer-assisted semen analysis. Acta Vet. Hung..

[36] Durairajanayagam D., Singh D., Agarwal A., Henkel R. (2021). Causes and consequences of sperm mitochondrial dysfunction. Andrologia.

[37] Solaini G., Sgarbi G., Lenaz G., Baracca A. (2007). Evaluating mitochondrial membrane potential in cells. Biosci. Rep..

[38] Sabés-Alsina M., Lundeheim N., Johannisson A., López-Béjar M., Morrell J.M. (2019). Relationships between climate and sperm quality in dairy bull semen: A retrospective analysis. J. Dairy Sci..

[39] Akbarinejad V., Fathi R., Shahverdi A., Esmaeili V., Rezagholizadeh A., Ghaleno L.R. (2020). The Relationship of Mitochondrial Membrane Potential, Reactive Oxygen Species, Adenosine Triphosphate Content, Sperm Plasma Membrane Integrity, and Kinematic Properties in Warmblood Stallions. J. Equine Vet. Sci..

[40] Yeste M., Estrada E., Rocha L.G., Marin H., Rodriguez-Gil J.E., Miro’ J. (2015). Cryotolerance of stallion spermatozoa is related to ROS production and mitochondrial membrane potential rather than to the integrity of sperm nucleus. Andrology.

[41] Gadella B.M., Rathi R., Brouwers J.F., Stout T.A., Colenbrander B. (2001). Capacitation and the acrosome reaction in equine sperm. Anim. Reprod. Sci..

[42] Aitken R.J. (1995). Free radicals, lipid peroxidation and sperm function. Reprod. Fertil. Dev..

[43] Bielski B.H., Arudi R.L., Sutherland M.W. (1983). A study of the reactivity of HO_2_/O_2_- with unsaturated fatty acids. J. Biol. Chem..

[44] Ortega Ferrusola C., González Fernández L., Morrell J.M., Salazar Sandoval C., Macías García B., Rodríguez-Martinez H., Tapia J.A., Peña F.J. (2009). Lipid peroxidation, assessed with BODIPY-C11, increases after cryopreservation of stallion spermatozoa, is stallion-dependent and is related to apoptotic-like changes. Reproduction.

[45] O’Flaherty C., Scarlata E. (2022). Oxidative stress and reproductive function: The protection of mammalian spermatozoa against oxidative stress. Reproduction.

[46] Catalán J., Yánez-Ortiz I., Torres-Garrido M., Ribas-Maynou J., Llavanera M., Barranco I., Yeste M., Miró J. (2024). Impact of Seminal Plasma Antioxidants on DNA Fragmentation and Lipid Peroxidation of Frozen-Thawed Horse Sperm. Antioxidants.

[47] Aitken R.J., Baker M.A. (2006). Oxidative stress, sperm survival and fertility control. Mol. Cell. Endocrinol..

[48] Griveau J.F., Le Lannou D. (1997). Reactive oxygen species and human spermatozoa: Physiology and pathology. Int. J. Androl..

[49] Drummen G.P., van Liebergen L.C., Op den Kamp J.A., Post J.A. (2002). C11-BODIPY(581/591), an oxidation-sensitive fluorescent lipid peroxidation probe: (micro)spectroscopic characterization and validation of methodology. Free Radic. Biol. Med..

[50] Neild D.M., Brouwers J.F., Colenbrander B., Agüero A., Gadella B.M. (2005). Lipid peroxide formation in relation to membrane stability of fresh and frozen thawed stallion spermatozoa. Mol. Reprod. Dev..

[51] Gibb Z., Lambourne S.R., Aitken R.J. (2014). The paradoxical relationship between stallion fertility and oxidative stress. Biol. Reprod..

[52] Aurich J., Kuhl J., Tichy A., Aurich C. (2020). Efficiency of Semen Cryopreservation in Stallions. Animals.

