# Supplementary material for: Repeatability of Selected Parameters Related to Stallion Sperm Quality and Cryotolerance
Source: Animals (Basel). 2025 Sep 26;15(19):2805. doi: 10.3390/ani15192805 (PMC12523851; doi:10.3390/ani15192805)
Supplement: Supplementary file 1 [file animals-15-02805-s001.zip › animals-3867107-supplementary.pdf]

Supplemental materials

Repeatability of Selected Parameters Related to Stallion Sperm Quality and Cryotolerance

by

Raffaele Boni, Raffaella Ruggiero, Felisia De Luca, Maria Lucia Serritella, Tommaso di Palma and Stefano Cecchini Gualandi

Table S1. Effect of stallion age on semen and sperm parameters in both fresh and frozen-thawed samples, using two extenders: HF-20 and INRA Freeze, with corresponding Pearson's correlation coefficients. Evaluated parameters include gel-free semen volume, sperm concentration, total spermatozoa per ejaculate, and physiological traits such as sperm kinetics, bioenergetics, and oxidative/nitrosative stress markers.

	Fresh sperm		**Frozen/thawed sperm**			
			**HF-20**		**INRA Freeze**	
	Age effect	*R*	*Age effect*	*R*	*Age effect*	*R*
Gel Free Volume	0.004**	+0.669***				
Sperm concentration	0.139	-0.510				
Spermatozoa per ejaculate	0.173	+0.515				
TM	0.001***	- 0.707***	0.232	-0.716***	0.165	-0.637**
PM	0.390	-0.565*	0.315	-0.675***	0.853	-0.561*
VCL	0.126	+0.469	0.062	+0.576*	0.001***	+0.715***
VSL	0.869	+0.325	0.358	+0.507	0.009**	+0.625**
VAP	0.468	+0.491	0.254	+0.515	0.003**	+0.659**
MMP	0.731	-0.247	0.096	+0.421	0.108	+0.202
LPO	0.007**	-0.259	0.045*	-0.535**	0.001***	-0.718***
H_2O_2 content	0.114	-0.196	0.003**	-0.552**	0.571	-0.261
NO content	0.100	-0.250	0.051	-0.407	0.017*	-0.506**

Total motility (TM), progressive motility (PM), curvilinear velocity (VCL), straight-line velocity (VSL), and average path velocity (VAP), mitochondrial membrane potential (MMP), lipid peroxidation (LPO), hydrogen peroxide (H_2O_2), and nitric oxide (NO).

* ($p \leq 0.05$), ** ($p \leq 0.01$), *** ($p \leq 0.001$).

Table S2. Effect of stallion age on sperm freezability, expressed as the ratio of thawed to fresh values, with corresponding Pearson correlation coefficients. Assessed parameters include motility kinetics, bioenergetics, and oxidative/nitrosative stress markers in sperm samples cryopreserved using two different freezing extenders (HF-20 and INRA Freeze®).

	**HF-20**		**INRA Freeze**	
	**Age effect p=**	**R**	**Age effect p=**	***R***
TM	0.257	-0.684***	0.179	-0.615**
PM	0.181	-0.655**	0.697	-0.517
VCL	0.285	+0.530	0.020*	+0.599**
VSL	0.461	+0.513	0.068	+0.559*
VAP	0.451	+0.521	0.058	+0.566*
MMP	0.730	+0.440	0.706	+0.366
LPO	0.702	-0.484	0.517	-0.577
H_2O_2 content	0.392	-0.522	0.901	-0.324
NO content	0.677	-0.310	0.982	-0.242

Total motility (TM), progressive motility (PM), curvilinear velocity (VCL), straight-line velocity (VSL), and average path velocity (VAP), mitochondrial membrane potential (MMP), lipid peroxidation (LPO), hydrogen peroxide (H_2O_2), and nitric oxide (NO).

* ($p \leq 0.05$), ** ($p \leq 0.01$).

Table S3. Semen and fresh sperm parameters in stallions grouped by age. Assessed parameters include semen traits, motility kinetics, bioenergetics, and oxidative/nitrosative stress markers.

		**≤ 10 years old mean ± SD**	**> 10 years old mean ± SD**	**p=**
Stallions	n.	5	6	
Age	years	6.2 ± 2.2	16.5 ± 1.6	0.001***
Gel Free Volume	mL	35.7 ± 21.5	52.5 ± 27.4	0.026*
Sperm concentration	x 10^6	357 ± 162	237 ± 156	0.019*
Spermatozoa per ejaculate	x 10^9	12.4 ± 8.5	12.4 ± 11.6	0.988
TM	%	88.3 ± 7.6	78.2 ± 14.8	0.010**
PM	%	35.6 ± 10.6	30.4 ± 10.7	0.101
VCL	μm s^{-1}	91.7 ± 16.6	92.0 ± 16.7	0.958
VSL	μm s^{-1}	36.0 ± 7.2	36.9 ± 6.8	0.697
VAP	μm s^{-1}	49.0 ± 9.8	50.9 ± 9.4	0.521
MMP	J_0B/J_0A	14.3 ± 9.0	12.9 ± 9.5	0.595
LPO	$C_0A/(C_0A+C_0B)$	12.4 ± 4.5	11.4 ± 3.6	0.379
H_2O_2 content	a.u.	3.0 ± 1.4	2.9 ± 1.4	0.646
NO content	a.u.	3.1 ± 1.4	2.2 ± 0.9	0.006**

Total motility (TM), progressive motility (PM), curvilinear velocity (VCL), straight-line velocity (VSL), and average path velocity (VAP), mitochondrial membrane potential (MMP), lipid peroxidation (LPO), hydrogen peroxide (H_2O_2), and nitric oxide (NO). a,b ($p \leq 0.05$), A,B ($p \leq 0.01$).

Table S4. Post-thaw sperm parameters in stallions grouped by age. Assessed parameters include motility kinetics, bioenergetics, and oxidative/nitrosative stress markers in samples cryopreserved with two different freezing extenders (HF-20 and INRA Freeze®).

		**HF-20**		**INRA Freeze**	
		**≤ 10 years old mean ± SD**	**> 10 years old mean ± SD**	**≤ 10 years old mean ± SD**	**> 10 years old mean ± SD**
TM	%	44.6 ± 17.7	39.4 ± 21.6	48.5 ± 15.4	40.3 ± 22.1
PM	%	9.4 ± 8.5	13.1 ± 13.3	9.0 ± 7.8	10.3 ± 8.6
VCL	$\mu m\ s^{-1}$	29.4 ± 11.0 ^{a}	39.4 ± 18.1 ^{b}	29.2 ± 9.4 ^{A}	41.9 ± 13.9 ^{B}
VSL	$\mu m\ s^{-1}$	13.8 ± 6.5	17.9 ± 8.9	13.1 ± 5.3 ^{a}	18.0 ± 6.7 ^{b}
VAP	$\mu m\ s^{-1}$	17.4 ± 6.9	22.1 ± 9.9	16.9 ± 5.7 ^{A}	22.9 ± 7.7 ^{B}
MMP	J_0B/J_0A	6.6 ± 4.0	8.0 ± 5.0	7.1 ± 10.6	8.4 ± 4.8
LPO	$C_0A/(C_0A+C_0B)$	14.1 ± 5.2	13.6 ± 6.5	16.4 ± 6.5	14.5 ± 5.1
H_2O_2 content	a.u.	1.8 ± 1.5 ^{a}	3.4 ± 3.1 ^{b}	3.4 ± 2.2	3.9 ± 2.5
NO content	a.u.	2.3 ± 1.9	2.1 ± 0.7	4.8 ± 3.2	3.5 ± 2.2

Total motility (TM), progressive motility (PM), curvilinear velocity (VCL), straight-line velocity (VSL), and average path velocity (VAP), mitochondrial membrane potential (MMP), lipid peroxidation (LPO), hydrogen peroxide (H_2O_2), and nitric oxide (NO). a,b ($p \leq 0.05$), A,B ($p \leq 0.01$).

Figure S1. Typical emission spectral diagrams recorded in stallion sperm loaded with different fluorochromes and their respective positive controls. Panel A reports the JC1 emission spectrum in control spermatozoa and after incubation with 2μM CCCP supplementated together with JC1 (CCCP was kept in the sample up to spectrofluorometer read). **Panel B** reports the C11-Bodipy$^{581\text{-}591}$ emission peak in C11- Bodipy$^{581\text{-}591}$-stained control spermatozoa and in spermatozoa treated with 1% OxMix (1) 1 hours before C11- Bodipy$^{581\text{-}591}$ staining. **Panel C** reports the H_2DCFDA emission spectrum in H_2DCFDA-stained control spermatozoa and after incubation with 1% OxMix together with H_2DCFDA. **Panel D** reports DAF emission spectrum in DAF-stained control spermatozoa and after incubation with 50 μM sodium nitroprusside (SNP) for 30 min after DAF loading.

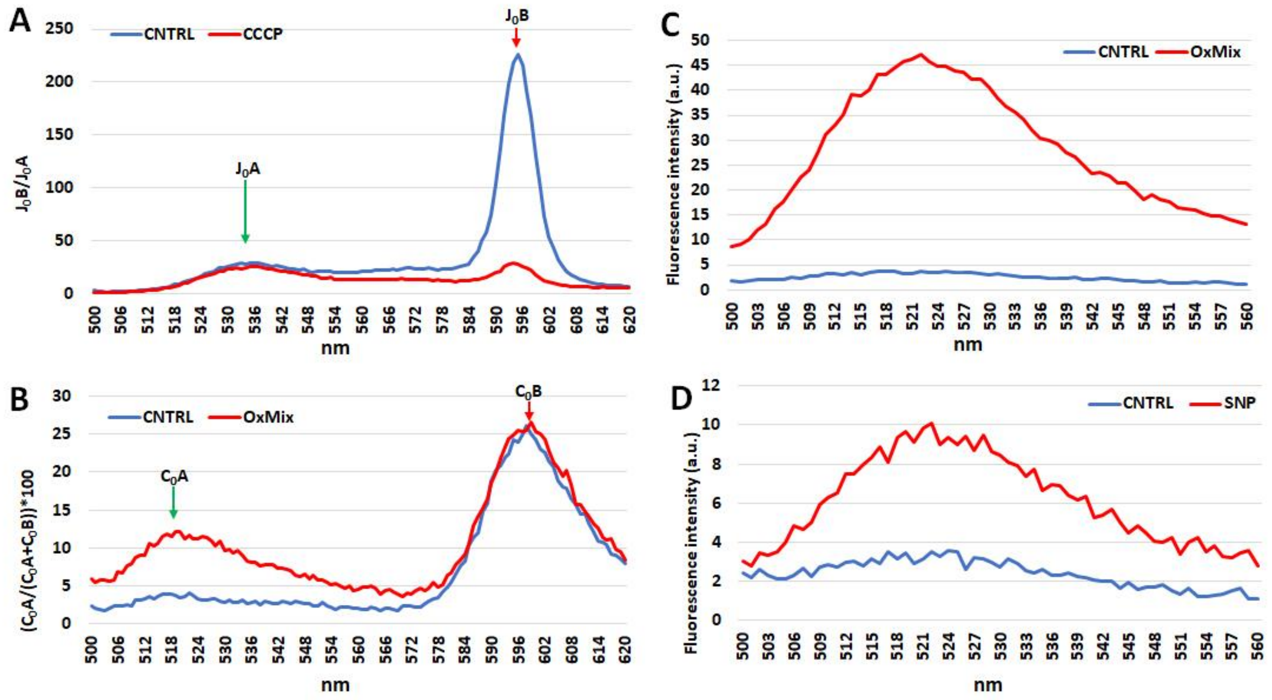

(1) OxMix solution contains 2 mM menadione, 0.25 mM hydrogen peroxide and 1.8 mM copper sulphate.
Di Palma et al., Zygote 2020 doi: 10.1017/S096719942000012X
Orhan et al., Toxicology in vitro 2006 doi:10.1016/j.tiv.2005.12.012

Figure S2. Principal component analysis (PCA) was performed on all kinetic parameters - Total Motility (TM), Progressive Motility (PM), Curvilinear Velocity (VCL), Straight-Line Velocity (VSL), and Average Path Velocity (VAP) - together with bioenergetic variables (mitochondrial membrane potential, MMP) and oxidative/nitrosative stress markers (lipid peroxidation, LPO; hydrogen peroxide, ROS; and nitric oxide, NO) in frozen-thawed semen from 11 Salernitano stallions using two different freezing extenders. The figure includes the path diagram, scree plot, component loadings, component characteristics, and correlation matrix, as well as the results of the Chi-square test, Bartlett's test, and the Kaiser-Meyer-Olkin (KMO) test.

Path diagram

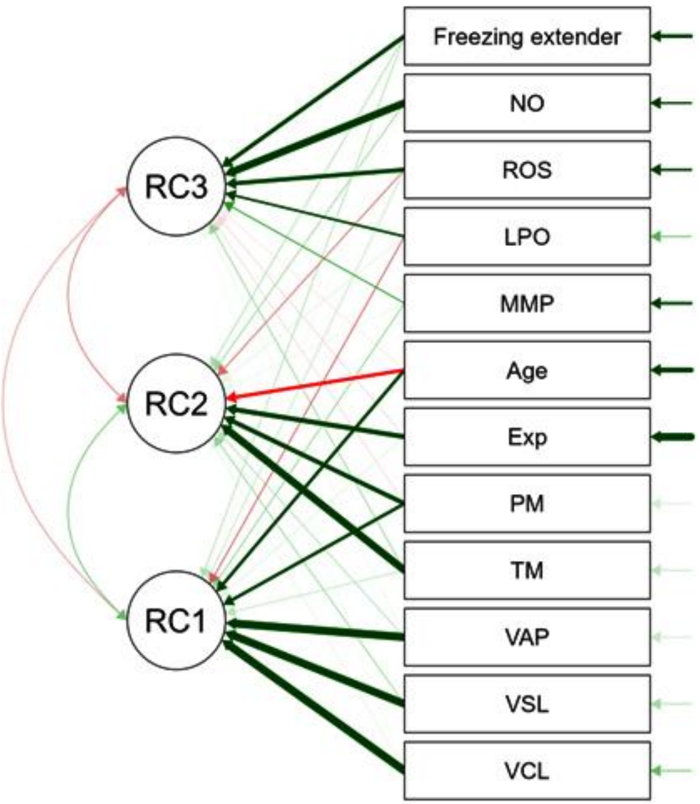

Scree plot

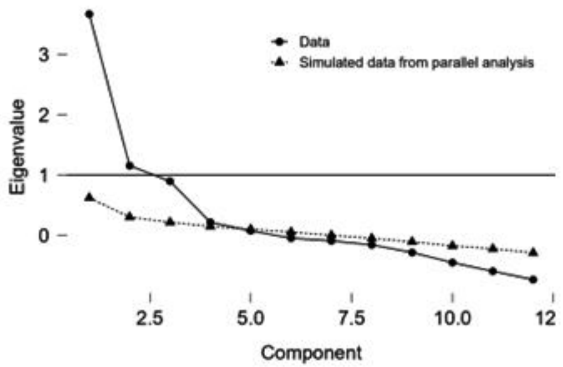

Component loadings

	RC1	RC2	RC3	Uniqueness
VCL	0.970			0.054
VAP	0.965			0.042
VSL	0.944			0.066
PM	0.570	0.669		0.092
Age	0.569	-0.571		0.457
TM		0.911		0.166
Exp		0.697		0.497
NO			0.912	0.185
ROS			0.647	0.532
Freezing extender			0.642	0.600
LPO			0.485	0.716
MMP				0.918

Note. Applied rotation method is promax.

Component characteristics

	Unrotated solution			Rotated solution		
	Eigenvalue	Proportion var.	Cumulative	SumSq. Loadings	Proportion var.	Cumulative
Component 1	3.924	0.327	0.327	3.524	0.294	0.294
Component 2	1.955	0.163	0.490	2.184	0.182	0.476
Component 3	1.796	0.150	0.640	1.968	0.164	0.640

Kaiser-Meyer-Olkin Test

	MSA
Overall MSA	0.498
TM	0.360
PM	0.640
VCL	0.551
VSL	0.515
VAP	0.510
MMP	0.194
LPO	0.387
ROS	0.472
NO	0.451
Exp	0.378
Age	0.622
Freezing extender	0.615

Chi-squared Test

	Value	df	p
Model	871.925	33	< .001

Component correlations

	Component 1	Component 2	Component 3
Component 1	1.000	0.162	-0.108
Component 2	0.162	1.000	-0.149
Component 3	-0.108	-0.149	1.000

Bartlett's Test

X^2	df	p
2799.528	66.000	< .001
